# Reply to “Potential confounders in linking elevated S100A8/A9 to left ventricular dysfunction in septic shock patients”

**DOI:** 10.1186/s13054-023-04789-9

**Published:** 2024-01-02

**Authors:** Gabriel Jakobsson, Henrik Andersson, Michelle Chew, Alexandru Schiopu

**Affiliations:** 1 Department of Translational Medicine, Lund University, Lund, Sweden; 2 Department of Anaesthesia and Intensive Care, Biomedical and Clinical Sciences, Linköping University, Linköping, Sweden; 3 Cardiac Inflammation Research Group, Clinical Research Center, 91:12, Jan Waldenströms Gata 35, 21 428 Malmö, Sweden; 4 Department of Internal Medicine, Skane University Hospital, Lund, Sweden; 5 Nicolae Simionescu Institute of Cellular Biology and Pathology, Bucharest, Romania

We would like to address the comments by Honoré et al*.* [1] referring to our recently published paper “Therapeutic S100A8/A9 blockade inhibits myocardial and systemic inflammation and mitigates sepsis‑induced myocardial dysfunction” [2].

In their commentary, Honoré et al*.* highlighted the importance of renal replacement therapy (RRT) as a potential factor that might have impacted the measurement of plasma S100A8/A9 in our study. Since the molecular weight of the S100A8/A9 heterodimer (24 kDa) is lower than the cutoff value of the extracorporeal purification membrane (35–40 kDa), the use of RRT might partly remove S100A8/A9 from the circulation, leading to falsely low values of the protein in plasma. The authors of the comment argue that this might have led to erroneous conclusions regarding the positive relationship between elevated plasma S100A8/A9 and the development of sepsis-induced myocardial dysfunction (SIMD) demonstrated by our study [2]. The concern regarding the influence of RRT on biomarker values in the context of sepsis is not specific for S100A8/A9 and has previously been expressed by Honoré et al*.* in reference to other studies [3].

We agree that biomarker washout by RRT is an important factor that might impact both clinical studies and clinical practice, regardless of the initial pathology leading to renal failure. However, we would like to stress that in our study, S100A8/A9 was measured in plasma collected within 12 h from admission to the intensive care unit (ICU), as mentioned in the Methods section [2]. At this early time point, none of the sepsis patients included in the cohort had received RRT. Subsequently, the results of our study showing that plasma S100A8/A9 is elevated in severe sepsis patients with SIMD have not been impacted by the use of RRT and remain valid.

## Data Availability

Not applicable.

## Abbreviations

RRT: Renal replacement therapy
SIMD: Sepsis-induced myocardial dysfunction
ICU: Intensive care unit
